# CELF significantly reduces milling requirements and improves soaking effectiveness for maximum sugar recovery of Alamo switchgrass over dilute sulfuric acid pretreatment

**DOI:** 10.1186/s13068-019-1515-7

**Published:** 2019-07-10

**Authors:** Abhishek S. Patri, Laura McAlister, Charles M. Cai, Rajeev Kumar, Charles E. Wyman

**Affiliations:** 1 Department of Chemical and Environmental Engineering, Bourns College of Engineering, University of California, Riverside, 900 University Ave, Riverside, CA 92521 USA; 2 BioEnergy Science Center (BESC), Oak Ridge National Laboratory (ORNL), Oak Ridge, TN 37831 USA; 3 Center for Environmental Research and Technology (CE-CERT), Bourns College of Engineering, University of California, Riverside, 1084 Columbia Ave, Riverside, CA 92507 USA; 4 Center for Bioenergy Innovation (CBI), Oak Ridge National Laboratory (ORNL), Oak Ridge, TN 37831 USA

**Keywords:** Biomass, Pretreatment, Soaking, Enzymatic hydrolysis, Dilute acid, Tetrahydrofuran, Size reduction

## Abstract

**Background:**

Pretreatment is effective in reducing the natural recalcitrance of plant biomass so polysaccharides in cell walls can be accessed for conversion to sugars. Furthermore, lignocellulosic biomass must typically be reduced in size to increase the pretreatment effectiveness and realize high sugar yields. However, biomass size reduction is a very energy-intensive operation and contributes significantly to the overall capital cost.

**Results:**

In this study, the effect of particle size reduction and biomass presoaking on the deconstruction of Alamo switchgrass was examined prior to pretreatment by dilute sulfuric acid (DSA) and Co-solvent Enhanced Lignocellulosic Fractionation (CELF) at pretreatment conditions optimized for maximum sugar release by each pretreatment coupled with subsequent enzymatic hydrolysis. Sugar yields by enzymatic hydrolysis were measured over a range of enzyme loadings. In general, DSA successfully solubilized hemicellulose, while CELF removed nearly 80% of Klason lignin from switchgrass in addition to the majority of hemicellulose. Presoaking and particle size reduction did not have a significant impact on biomass compositions after pretreatment for both DSA and CELF. However, presoaking for 4 h slightly increased sugar yields by enzymatic hydrolysis of DSA-pretreated switchgrass compared to unsoaked samples, whereas sugar yields from enzymatic hydrolysis of CELF solids continued to increase substantially for up to 18 h of presoaking time. Of particular importance, DSA required particle size reduction by knife milling to < 2 mm in order to achieve adequate sugar yields by subsequent enzymatic hydrolysis. CELF solids, on the other hand, realized nearly identical sugar yields from unmilled and milled switchgrass even at very low enzyme loadings.

**Conclusions:**

CELF was capable of achieving nearly theoretical sugar yields from enzymatic hydrolysis of pretreated switchgrass solids without size reduction, unlike DSA. These results indicate that CELF may be able to eliminate particle size reduction prior to pretreatment and thereby reduce overall costs of biological processing of biomass to fuels. In addition, presoaking proved much more effective for CELF than for DSA, particularly at low enzyme loadings.

**Electronic supplementary material:**

The online version of this article (10.1186/s13068-019-1515-7) contains supplementary material, which is available to authorized users.

## Background

Biofuels derived from lignocellulosic biomass have the potential to substantially reduce greenhouse emissions and dependence on vulnerable and depletable fossil fuel resources [1, 2]. Switchgrass (*Panicum virgatum*) is a leading candidate as an effective bioenergy feedstock due to its perennial nature, high productivity, and soil restoration properties [3–5]. Switchgrass is mostly composed of carbohydrates and lesser amounts of lignin, with minor contributions from ash, extractives, and protein [6–8]. Cellulose and hemicellulose are the carbohydrates of primary interest for biological production of biofuels, as they can be broken down into five and six carbon sugars that microorganisms can ferment to ethanol with high yields. However, due to the complex nature of plant cell walls, pretreatment is typically required prior to enzymatic and biological conversion to expose carbohydrates from the lignin shield [9]. Various pretreatments that can be broadly categorized as mechanical, thermal, chemical, or their combination have been developed over the years to overcome this recalcitrance to sugar release [10, 11]. Mechanical methods typically involve particle size reduction by milling to increase enzyme access to cell wall carbohydrates [12, 13]. Thermochemical pretreatments utilize chemical reagents, such as acids, bases, or solvents, at elevated temperatures to disrupt the cell wall structure and achieve greater access to carbohydrates [14]. Dilute sulfuric acid (DSA) pretreatment is a research and commercial benchmark that solubilizes hemicellulose to sugars with high yields and increases digestibility of pretreated biomass, although high enzyme loadings are required for the latter to achieve satisfactory sugar yields [15]. Recent advanced pretreatment technologies, such as ammonia fiber expansion (AFEX), organosolv, ionic liquid, and sulfite pretreatments, have made strides in improving cellulose digestibility and increasing enzymatic sugar yields from pretreated biomass [16–19]. Co-solvent Enhanced Lignocellulosic Fractionation (CELF) is a recently developed advanced pretreatment that utilizes dilute acid in a miscible mixture of tetrahydrofuran (THF) and water to recover about 80–90% of the lignin and > 95% hemicellulose sugars in solution and achieve nearly theoretical sugar yields from the glucan and hemicellulose left in the resulting carbohydrate-rich solids at low enzyme loadings [20].

Several challenges are yet to be addressed before biomass-derived fuels can be considered cost competitive [21]. For one, because pretreatment is one of the most expensive single unit operations in a biomass processing plant [22], pretreatment cost reductions would be a significant step to lowering the cost of cellulosic biofuels. Lignocellulosic biomass particle size reduction and presoaking prior to pretreatment are typically needed to increase biomass surface area and effectively distribute acid (or other catalyst) throughout the biomass solids, respectively [23, 24] so that high sugar yields can be achieved from pretreatment combined with subsequent enzymatic hydrolysis [25]. Presoaking of biomass with the reaction ingredients at ambient temperatures prior to thermochemical pretreatment has also been shown to increase biomass wetting and improve inter-particle diffusion of acid catalysts [26–28]. However, these additional steps increase overall capital and operating costs for making biomass-derived fuels. Particle size reduction, in particular, can require intensive energy inputs [29–32], and reducing milling or eliminating it altogether has been proposed to lower pretreatment costs. In this study, DSA and CELF pretreatment temperatures and times were varied to maximize overall release of glucan and xylan from each pretreatment coupled with subsequent enzymatic hydrolysis of milled and presoaked Alamo switchgrass. At these maximum sugar release conditions, the impacts of biomass presoaking and particle size reduction by knife milling were assessed for both DSA and CELF pretreatment of Alamo switchgrass. Solids after both pretreatments were analyzed for compositional differences at varying presoaking times and particle sizes. Furthermore, sugar yields from enzymatic hydrolysis of pretreated solids were compared over a range of enzyme loadings to determine the impact of presoaking and knife milling on biomass sugar release following DSA and CELF pretreatments.

## Results and discussion

### Maximizing overall glucose and xylose sugar yields from switchgrass by DSA and CELF pretreatments followed by enzymatic hydrolysis

**Fig. 1:**
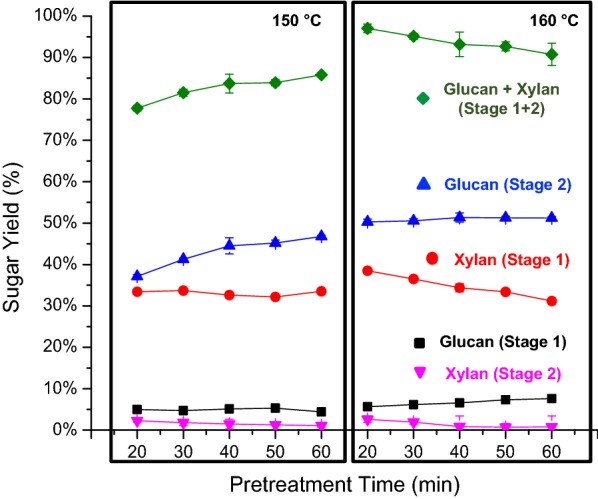
Effect of pretreatment time at 150 °C and 160 °C on glucan, xylan, and total glucan plus xylan yields from dilute sulfuric acid (DSA) pretreatment (Stage 1) of switchgrass, enzymatic hydrolysis of the pretreated solids (Stage 2), and the two stages combined. Stage 1 reaction conditions: solids loading of 7.5 wt% with an acid loading of 0.5 wt%. Stage 2 enzymatic hydrolysis was performed on pretreated solids at a 10 g/L glucan loading with 65 mg of Accellerase^®^ 1500 protein/g glucan in unpretreated switchgrass

**Fig. 2:**
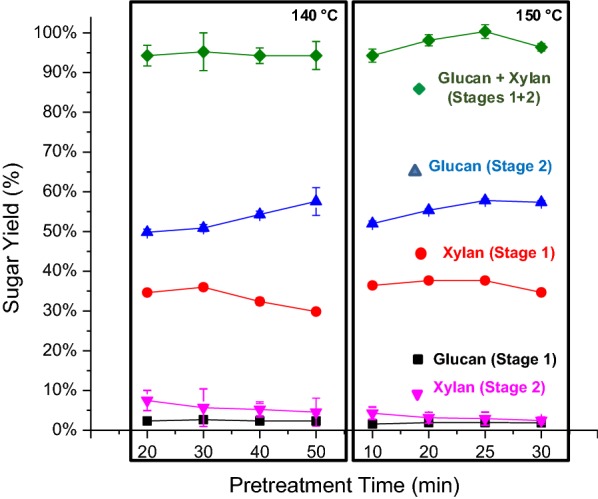
Effect of pretreatment time at 140 °C and 150 °C on glucan, xylan, and total glucan plus xylan yields from CELF pretreatment (Stage 1) of switchgrass, enzymatic hydrolysis of the pretreated solids (Stage 2), and the two stages combined. Stage 1 reaction conditions: solids loading of 7.5 wt% with an acid loading of 0.5 wt % based on liquid weight, THF/water mass ratio-0.889:1. Stage 2 enzymatic hydrolysis was performed on pretreated solids at a 10 g/L glucan loading with 65 mg of Accellerase^®^ 1500 protein/g glucan in unpretreated switchgrass

Switchgrass was subjected to DSA and CELF pretreatments to identify conditions that maximized glucan plus xylan yields from each pretreatment coupled with subsequent enzymatic hydrolysis of the pretreated solids. DSA pretreatments were performed at 150 and 160 °C, and CELF pretreatments were at 140 and 150 °C, as these temperature ranges have previously been shown to be optimum for switchgrass [33] and corn stover [34], respectively. The reactions at each temperature were carried out over a range of times to be sure that differences in biomass sources did not alter the time to achieve the highest sugar yields. The liquid hydrolyzates from both pretreatments were analyzed for total dissolved glucose and xylose including gluco- and xylo-oligomers. The pretreatment step alone was termed Stage 1, and the sugars released were expressed in terms of equivalent glucan and xylan by taking into account the water added during hydrolysis (see Additional file 1: Figure S1). The pretreated solids were then subjected to enzymatic hydrolysis at a high cellulase loading of 65 mg protein/g glucan in unpretreated switchgrass to determine the maximum possible sugar release at each pretreatment condition. The enzymatic hydrolysis step was termed Stage 2 (see Additional file 1: Figure S1). Figures 1 and 2 summarize the trends in glucan and xylan released in Stages 1 and 2 alone, as well as the combined glucan and xylan yields from Stage 1 + 2 together.

As expected, increasing time during both pretreatments initially increased Stage 1 xylan release as more xylan from the hemicellulose fraction was solubilized by the acid catalyst. However, at pretreatment times > 30 min, significant amounts of xylose were dehydrated to furfural, which reduced the maximum possible xylose yield. Sugar dehydration products, furfural, and 5-hydroxymethylfurfural (HMF) concentrations were quantified for both pretreatments using HPLC (Additional file 1: Table S1). At maximum sugar release conditions for both DSA and CELF, furfural and HMF concentrations in pretreatment hydrolyzates were below HPLC detection limit (< 0.1 g/L). Glucan, on the other hand, was largely conserved in the solid in Stage 1 for both pretreatments, as the pretreatment conditions were not harsh enough to solubilize significant amounts of crystalline cellulose or degrade much of the dissolved glucan. The small amount of glucan released during pretreatment, termed Stage 1 glucan, was likely mostly from hemicellulose and the amorphous portion of cellulose and was robust enough to suffer little degradation at the pretreatment conditions applied [35]. Increasing pretreatment time made biomass more susceptible to enzymatic breakdown by hydrolysis of pretreated solids, as illustrated by the increase in Stage 2 glucan release with increasing pretreatment time. Since the Accellerase^®^ 1500 cellulase cocktail contains some hemicellulases and as well as auxiliary enzymes [36], residual xylan in pretreated solids was also solubilized during enzymatic hydrolysis and reported as Stage 2 xylan. The conditions that maximized sugar release for DSA and CELF pretreatments were 160 °C, 20 min and 150 °C, 25 min, respectively, demonstrating that CELF reduced the temperature needed to achieve maximum sugar yields by 10 °C from that needed for DSA (Figs. 1 and 2). The sugars solubilized during pretreatment at these conditions yielded liquids containing 2.4 g/L glucose, 20.2 g/L xylose, and 2.5 g/L arabinose in DSA hydrolyzate and 3.1 g/L glucose, 21 g/L xylose, and 2.0 g/L arabinose in CELF hydrolyzate.

**Fig. 3:**
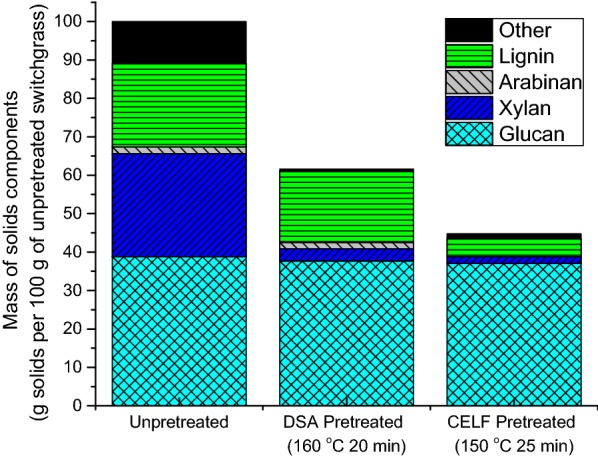
Tracking mass of glucan, xylan, and lignin left in the solids produced by DSA and CELF pretreatments at conditions optimized to maximize total overall glucan plus xylan yields. The values shown are based on the content of each component in 100 g of switchgrass before pretreatment. Reaction conditions: DSA at 160 °C for 20 min with 0.5 wt% sulfuric acid, and CELF at 150 °C for 25 min with 0.5 wt % sulfuric acid and a 0.889:1 THF/water mass ratio

The compositions of pretreated solids prepared at all pretreatment conditions were analyzed to determine the fate of components in the solids left by pretreatment. The mass of each component in solids produced by application of the maximum sugar recovery pretreatment conditions for both DSA and CELF pretreatments was then adjusted to a basis of 100 g of unpretreated switchgrass, as shown in Fig. 3. For both pretreatments at maximum sugar recovery conditions, most of the hemicellulose sugars (mostly xylan) were solubilized during pretreatment, in agreement with the previous results for both of these pretreatments [34]. Glucan was largely conserved in both pretreatments as expected.

The major difference between DSA and CELF-pretreated solids was the amount of lignin left in pretreated solids. While DSA removed roughly 14 wt% of Klason lignin (K-lignin), CELF removed 77 wt% of K-lignin at optimized conditions. This greater degree of delignification is encouraging as lignin has been shown to be a major contributor to biomass recalcitrance [37]. At conditions optimized for maximum sugar recovery, the solids produced by DSA pretreatment contained 65% glucan, 4% xylan, and 32% K-lignin. CELF-pretreated solids, on the other hand, contained 86% glucan, 4% xylan, and 11% K-lignin at optimized conditions, consistent with enhanced lignin removal by CELF. Compositional analyses on solids resulting from more severe CELF pretreatments revealed that more lignin was removed at higher severities. However, a drawback was that more xylan was lost to dehydration products.

### Effects of presoaking and particle size reduction on compositions of Alamo switchgrass solids pretreated by DSA and CELF

To understand the effect of presoaking on DSA and CELF at the previously optimized pretreatment conditions, unpretreated switchgrass that was knife milled to < 1 mm was soaked for 4 and 18 h at 4 °C prior to pretreatment. These solids were compared to samples that were not soaked prior to pretreatment. The effect of particle size reduction on switchgrass was determined by presoaking unmilled and knife-milled biomass for 18 h at 4 °C before pretreatment. Additional file 1: Figure S2 shows images of unmilled switchgrass and switchgrass knife milled through sieve sizes of 2 mm and 1 mm.

The masses of major components of Alamo switchgrass solids after DSA and CELF pretreatments at varying presoaking times and particle sizes are listed in Table 1. While expected results of xylan removal by both pretreatments and delignification during CELF were observed, minimal differences were observed in the masses of major components as a function of presoaking times for both pretreatments. However, unsoaked DSA-pretreated switchgrass contained slightly more glucan in the solids compared to soaked samples, implying that pretreatment acid was not able to fully reach cellulose without soaking, thus resulting in less glucan removal. On the other hand, CELF-pretreated switchgrass that was soaked for 4 and 18 h prior to pretreatment was slightly more delignified than samples that were not soaked prior to CELF. No major compositional differences were observed in solids produced by pretreatment of the range of particle sizes. As expected, both pretreatments removed hemicellulose from the solids, and CELF removed the majority of lignin from solid biomass. These results show that CELF removed nearly 80% of the lignin from switchgrass even without presoaking or particle size reduction prior to pretreatment.

**Table 1:** Masses of glucan, xylan, and lignin in solids for unpretreated switchgrass and following DSA and CELF pretreatments of switchgrass for varying presoaking times and particle sizes

Pretreatment	Presoaking time^a^	Particle size^b^	Glucan (g)	Xylan (g)	Lignin (g)
Unpretreated	–	–	38.8	26.8	21.7
DSA	0 h	< 1 mm	37.5	2.8	17.7
DSA	4 h	< 1 mm	36.3	2.7	17.8
DSA	18 h	< 1 mm	35.8	2.9	17.6
CELF	0 h	< 1 mm	37.4	2.7	4.6
CELF	4 h	< 1 mm	36.8	2.3	4.0
CELF	18 h	< 1 mm	37.0	1.7	3.8
DSA	18 h	Unmilled	36.0	2.7	18.6
DSA	18 h	< 2 mm	35.9	2.7	17.6
DSA	18 h	< 1 mm	35.8	2.9	17.6
CELF	18 h	Unmilled	37.2	1.6	4.1
CELF	18 h	< 2 mm	36.8	1.7	4.0
CELF	18 h	< 1 mm	37.0	1.7	3.8

^a^Dilute sulfuric acid (DSA) samples were presoaked in 0.5 wt% sulfuric acid, and Co-solvent Enhanced Lignocellulosic Fractionation (CELF) samples were presoaked in 0.5 wt% sulfuric acid at a 0.889:1 THF/water mass ratio, both at 4 °C

^b^Particle size reduction achieved by knife milling

### Effect of presoaking on enzymatic hydrolysis of DSA- and CELF-pretreated Alamo switchgrass solids

**Fig. 4:**
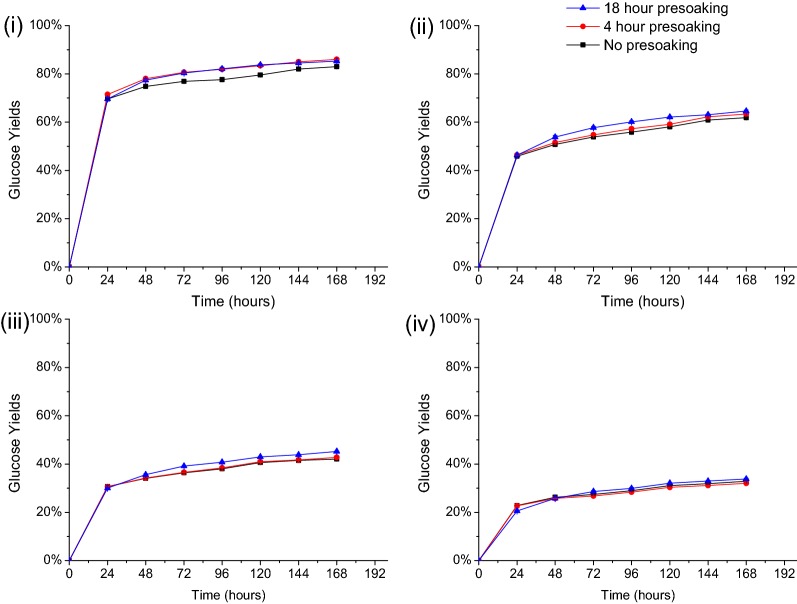
Effect of DSA presoaking time on glucose yields from hydrolysis of pretreated solids by Accellerase 1500 cellulase at loadings of **i** 65 mg, **ii** 15 mg, **iii** 5 mg, and **iv** 2 mg cellulase protein/g glucan in unpretreated switchgrass. All DSA pretreatments were performed at 160 °C for 20 min with 0.5 wt% sulfuric acid to 7.5 wt% solid loadings of switchgrass knife milled to < 1 mm

Solids left after DSA and CELF pretreatments with presoaking for 4 and 18 h at 4 °C and without presoaking were hydrolyzed with Accellerase^®^ 1500 cellulase at loadings of 65, 15, 5, and 2 mg protein/g glucan in unpretreated solids. At the highest enzyme loading of 65 mg protein/g glucan, Fig. 4(i) points out that for DSA, presoaking for 4 h increased glucose yields by 3% (± 0.04%) at the end of 7 days of enzymatic hydrolysis compared to solids that were not presoaked. However, increasing presoaking to 18 h did not affect glucose yields. Similar trends were observed at the lower enzyme loadings, with the minor differences in yields between presoaking times of 4 and 18 h indicating that 4 h of presoaking prior to DSA pretreatment was sufficient to realize virtually maximum glucan release.

**Fig. 5:**
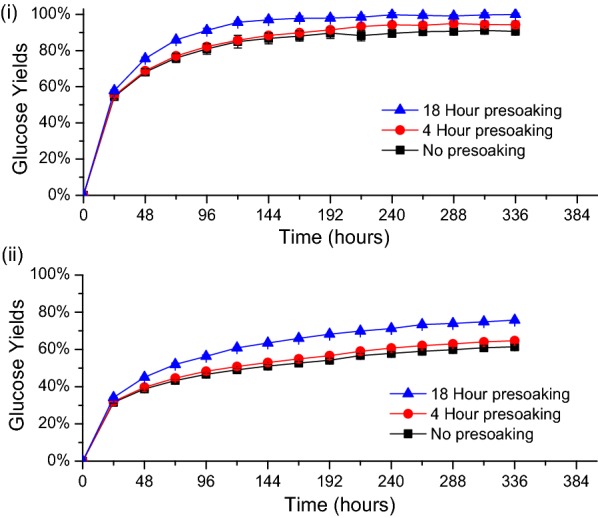
Effect of presoaking time on glucose yields from enzymatic hydrolysis of CELF pretreated by Accellerase 1500 cellulase at loadings of **i** 5 mg protein/g glucan in unpretreated switchgrass and **ii** 2 mg protein/g glucan in unpretreated switchgrass. All CELF pretreatments were performed at 150 °C for 25 min with 0.5 wt% sulfuric acid and a 0.889:1 THF/water mass ratio to 7.5 wt% solid loadings of switchgrass knife milled to < 1 mm

As previously shown [34], CELF produced highly digestible solids that were virtually completely hydrolyzed to glucose in 48 h at enzyme loadings of 65 and 15 mg protein/g glucan. Thus, enzyme loadings of 5 and 2 mg protein/g glucan were applied to more clearly show the effect of presoaking times on enzymatic hydrolysis of CELF switchgrass. Figure 5(i) shows that presoaking of switchgrass for 4 h prior to CELF increased glucose yields by 3% (± 0.53%) for hydrolysis over 2 weeks at an enzyme loading of 5 mg protein/g glucan compared to unsoaked switchgrass. Furthermore, presoaking switchgrass for 18 h increased glucose yields an additional 4% (± 0.72%) to reach 100% in 2 weeks, as also shown in Fig. 5(i). For the enzyme loading of 2 mg protein/g glucan in Fig. 5(ii), 18 h of presoaking prior to CELF increased glucose yields from 2 weeks of enzymatic hydrolysis by 14% (± 0.70%) compared to unsoaked switchgrass. However, presoaking for more than 18 h produced no change in glucose yields (data not shown).

The improved sugar yield from CELF switchgrass upon increasing presoaking time indicated better presoaking effectiveness of the CELF mixture. It is hypothesized that THF penetrated biomass more effectively than water alone could, improving solvent contact possibly due to its significantly lower surface tension compared to pure water, thus increasing sugar yields with increasing presoaking time [38, 39]. The results of a simple experiment to test this hypothesis show that THF required far less time to wet 0.25 g of milled switchgrass than DI water in a stemmed funnel. THF required 20 min, whereas DI water required 8 h. Furthermore, the solution of THF and water in the CELF mixture (0.889:1 THF/water by mass) took 3.5 h, which was half the time of DI water alone to wet switchgrass, suggesting the ability of the CELF mixture to better soak switchgrass and increase the effectiveness of presoaking prior to pretreatment.

### Effect of particle size reduction prior to DSA and CELF pretreatments on enzymatic hydrolysis of Alamo switchgrass

**Fig. 6:**
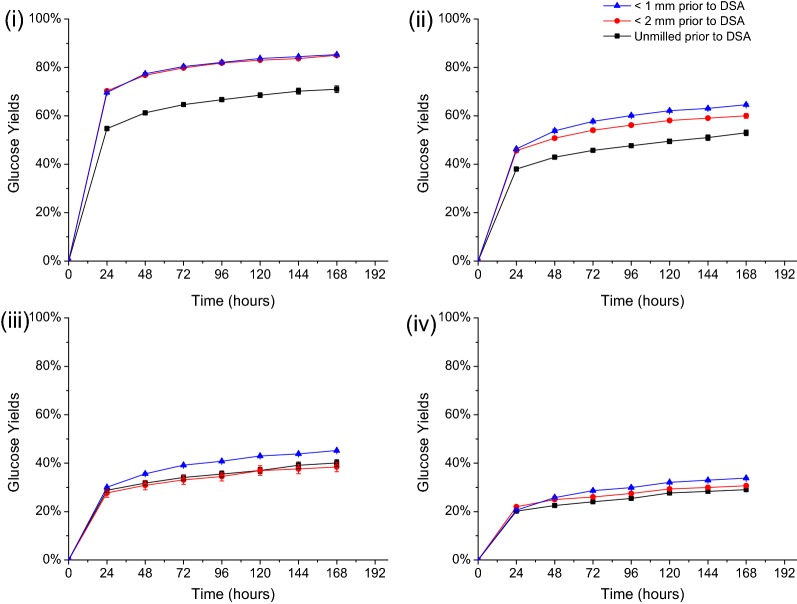
Effect of milling (particle size) on enzymatic glucose yields for pretreated solids prepared by DSA at Accellerase 1500 cellulase loadings of **i** 65 mg, **ii** 15 mg, **iii** 5 mg, and **iv** 2 mg cellulase protein/g glucan in unpretreated switchgrass. All DSA pretreatments were performed at 160 °C for 20 min with 0.5 wt% sulfuric acid on switchgrass that had first been presoaked for 18 h at 4 °C at a 7.5 wt% solids loading

Solids produced by CELF and DSA pretreatments of switchgrass that had been presoaked for 18 h with and without prior milling were hydrolyzed by Accellerase^®^ 1500 over a range of enzyme loadings. Figure 6 shows that milling significantly improved sugar yields from enzymatic hydrolysis of DSA-pretreated switchgrass solids. For example, glucose yields from DSA pretreatment of unmilled switchgrass were 14% (± 1.15%) lower than those from milled switchgrass even at a very high enzyme loading of 65 mg protein/g glucan (Fig. 6(i)). Furthermore, yields from enzymatic hydrolysis of solids produced by DSA pretreatment of unmilled switchgrass were lower at enzyme loadings of 15, 5, and 2 mg protein/g glucan compared to those from DSA on milled switchgrass. The sieve size used during milling, however, only had a slight effect on glucose yields from enzymatic hydrolysis of DSA-pretreated switchgrass.

**Fig. 7:**
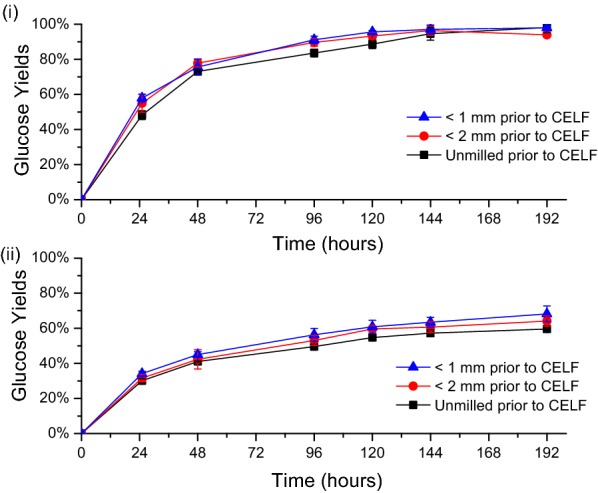
Effect of milling (particle size) on glucose yields from CELF-pretreated solids for enzymatic hydrolysis with Accellerase 1500 at cellulase loadings of **i** 5 mg and **ii** 2 mg cellulase protein/g glucan in unpretreated switchgrass. All CELF pretreatments were performed at 150 °C for 25 min with 0.5 wt% sulfuric acid and a 0.889:1 THF/water mass ratio for switchgrass that had first been presoaked for 18 h at 4 °C and a 7.5 wt% solids loading

Because CELF pretreatment achieved nearly theoretical yields from enzymatic hydrolysis at high enzyme loadings, only low loadings of 5 and 2 mg protein/g glucan were applied so the effects of particle size could be distinguished. As shown in Fig. 7, all samples were highly digestible after 8 days of hydrolysis even at these very low enzyme loadings. Furthermore, glucose yields from enzymatic hydrolysis of CELF-pretreated switchgrass were within standard deviation (± 0.83%) regardless of whether the switchgrass was milled or not, and the particle size from milling did not affect glucose yields. These results suggest that CELF is capable of achieving high sugar yields from switchgrass even without prior particle size reduction.

These results demonstrated that CELF pretreatment can remove a large portion of the lignin and hemicellulose without particle size reduction by knife milling. They also showed that milling has very little effect on glucose yields from enzymatic hydrolysis of CELF-pretreated solids. These outcomes are in stark contrast to those for DSA pretreatment for which particle size reduction to at least < 2 mm was required to achieve comparable sugar yields to CELF albeit at much greater loadings of expensive enzymes. Milling prior to pretreatment had a minor effect on the composition of solids produced by DSA pretreatment of switchgrass, implying that the increase in enzymatic hydrolysis yields with milling of DSA switchgrass resulted from enhanced micro-accessibility of cellulose [40] that can be improved by reducing cellulose crystallinity or degree of polymerization [41]. Because DSA solubilizes hemicellulose and increases cellulose accessibility without physically removing much of the lignin from biomass, it is likely that acid for DSA pretreatment does not effectively diffuse through the entire particle to contact all of the cellulose microfibrils and make them more micro-accessible to cellulolytic enzymes. For CELF pretreatment, on the other hand, the THF/water co-solvent solubilizes a large fraction of the lignin as well as hemicellulose to thus increase the glucan content in the pretreated solids. As lignin in plants coats cell wall polysaccharides and thereby impairs water access [42], removal of most of the lignin in addition to hemicellulose by CELF pretreatment of unmilled switchgrass could disintegrate the cell wall structure and allow acid catalyst to freely contact cellulose fibers.

CELF pretreatment has previously been demonstrated to reduce the amount of enzyme required to achieve high glucose yields and high ethanol titers from corn stover [20, 43]. This study showed that those findings apply to CELF pretreatment of switchgrass with 100% glucose yields achieved at enzyme loadings as low as 5 mg protein/g glucan. Because enzymes are a major contributor to the cost of cellulosic fuel production [44], realizing high sugar yields with much less enzyme can have a major impact on process economics [29, 30, 32]. In addition, the results presented here suggest that energy-intensive milling can be eliminated for CELF pretreatment of switchgrass but not for DSA pretreatment. Although elimination of particle size reduction could have significant commercial implications, the effect of higher solids loadings on the performance of CELF pretreatment and subsequent enzymatic hydrolysis must still be ascertained.

## Conclusions

Most biological operations for biomass conversion require particle size reduction prior to pretreatment to realize high sugar yields by subsequent enzymatic hydrolysis. Biomass is also presoaked prior to most pretreatments to provide adequate reactant contact. Thus, both particle size reduction and presoaking can increase reactant diffusion into the biomass particle. However, the high milling energy required to reduce particle size sufficiently to realize high yields is a significant contributor to processing costs, and extended presoaking increases processing times. In this study, the effect of presoaking times and particle size reduction by knife milling on DSA and CELF pretreatment of Alamo switchgrass was investigated. Biomass presoaking slightly increased glucose yields from enzymatic hydrolysis for DSA and CELF solids. Significantly, CELF pretreatment is shown to be capable of achieving high glucose yields from subsequent enzymatic hydrolysis of solids from CELF pretreatment of switchgrass without size reduction even at low enzyme loadings, in definite contrast to DSA. The latter results indicate that particle size reduction of biomass could be eliminated prior to CELF pretreatment without a reduction in sugar yields, thus potentially reducing processing costs for biofuels production.

## Methods

### Materials

Chopped senescent Alamo switchgrass (diameter = 0.35 ± 0.1 cm) was provided by Genera Energy Inc. (Vonore, TN). DuPont Industrial Biosciences (Palo Alto, CA) provided the Accellerase^®^ 1500 fungal cellulolytic enzyme cocktail used for enzymatic hydrolysis. The protein concentration was measured as 82 mg/ml by following the standard BCA method with bovine serum albumin as a standard [45]. Tetrahydrofuran (THF) was purchased from Fisher Scientific (Pittsburgh, PA).

### Milling and soaking

Knife milling was performed using a Wiley Mill (Model 4, Arthur H. Thomas Company, Philadelphia, PA) with a 1 mm or 2 mm particle size interior sieve. Prior to pretreatment, milled and unmilled switchgrass solids were soaked for times varying from 0 to 18 h in appropriate reaction ingredients (see “Pretreatment” section) in the pretreatment reactor at 4 °C in a refrigerator to minimize reaction during presoaking and minimize solvent evaporation.

To test the wetting properties of THF and water to switchgrass, 0.25 g of milled Alamo switchgrass (< 1 mm) was packed into a stemmed glass funnel, and the bottom was plugged with cheesecloth. The plugged end was then inserted into a conical tube containing 10 mL of DI water, pure THF, or a 0.889:1 mixture of THF/water by mass. A timer was started when the funnel stem first made contact with the liquid, and the timer was stopped when the biomass was completely soaked. The timer reading was recorded as the time taken to wet 0.25 g of milled Alamo switchgrass.

### Pretreatment

Pretreatments were performed in a 1 L Parr^®^ Hastelloy autoclave reactor (236HC Series, Parr Instruments Co., Moline, IL) equipped with a double-stacked pitch blade impeller rotating at 200 rpm. For DSA reactions, solutions were loaded with 0.5 wt% (based on liquid mass) sulfuric acid (Ricca Chemical Company, Arlington, TX), while for CELF reactions, THF (> 99% purity, Fisher Scientific, Pittsburgh, PA) was added to a 0.5 wt% sulfuric acid solution in water at a 0.889:1 THF to acidic water mass ratio. All reactions were maintained at reaction temperature (± 1 °C) by convective heating with a 4 kW fluidized sand bath (Model SBL-2D, Techne, Princeton, NJ). Sand bath temperature was maintained at twice the target pretreatment temperature to minimize heat up time. Heat up times were kept under 2 min for all pretreatments. The reaction temperature was directly measured by an in-line K-type thermocouple (Omega Engineering Inc., Stamford, Connecticut). Following pretreatment, solids were separated from the liquid by vacuum filtration at room temperature through glass fiber filter paper (Fisher Scientific, Pittsburgh, PA) and washed with room temperature deionized water until the filtrate pH reached neutral. The solids were carefully transferred to ziplock bags and weighed. Moisture content of the solids was determined by a halogen moisture analyzer (Model HB43, Mettler Toledo, Columbus, OH). Pretreatment hydrolyzate was collected for HPLC analysis of sugars and sugar dehydration products. Density of hydrolyzate was determined by measuring the mass of hydrolyzate contained in a 25-mL volumetric flask.

### Enzymatic hydrolysis

Enzymatic hydrolysis was performed per a NREL protocol [37] in triplicate in 125-mL Erlenmeyer flasks with a 50 g total working mass that contained 50 mM sodium citrate buffer (pH 4.9) to maintain the hydrolysis pH and 0.02% sodium azide to prevent microbial contamination together with enough pretreated solids to result in approximately 1 wt% glucan. Accellerase^®^ 1500 cellulase loadings for enzymatic hydrolysis were varied from 2 to 65 mg protein/g glucan in unpretreated biomass [31]. Enzyme loadings were based on unpretreated switchgrass so as not to penalize a pretreatment if it released more glucose in the pretreatment step. Enzymatic hydrolysis flasks were placed in a Multitron orbital shaker (Infors HT, Laurel, MD) set at 150 rpm and 50 **°**C and allowed to equilibrate for 1 h before enzyme addition. Homogenous samples of approximately 500 μL were collected at 4 h, 24 h, and every 24 h thereafter, into 2 mL centrifuge tubes (Fisher Scientific, Pittsburg, PA), and then centrifuged at 15,000 rpm for 10 min before analysis of the supernatant by HPLC.

### Analytical procedures

All chemical analyses followed Laboratory Analytical Procedures (LAPs) documented by the National Renewable Energy Laboratory (NREL, Golden, CO). Compositional analyses of unpretreated and pretreated switchgrass were according to the NREL protocol in triplicates [46]. Residual mass after quantification of carbohydrates and lignin, which includes ash, proteins, etc., was expressed as “Other.” Liquid samples along with appropriate calibration standards were analyzed on an HPLC (Waters Alliance e2695) equipped with a Bio-Rad Aminex^®^ HPX-87H column and RI detector (Waters 2414) with an eluent (5 mM sulfuric acid) flow rate at 0.6 mL/min. The chromatograms were integrated using an Empower^®^ 2 software package (Waters Co., Milford, MA).

### Calculations

After HPLC quantification, the following formulae were applied to calculate mass, volumes, enzyme loadings, and yields:

$${\text{Mass of sugar released in pretreatment hydrolyzate}} = {\text{Sugar concentration from HPLC}}*{\text{Volume of pretreatment hydrolyzate}}$$

$${\text{Volume of pretreatment hydrolyzate}} = \left( {{\text{Total reaction mass}} -\, \left( {{\text{Mass of wet pretreated solids}}*{\text{Moisture content}}} \right)} \right)/{\text{Hydrolyzate density}}$$

$${\text{Glucan yield after pretreatment}} = \left( {{\text{Mass of wet pretreated solids}}*\left( { 100 - {\text{Moisture content}}} \right)*\% \;{\text{of glucan in pretreated solids}}} \right)/\left( {{\text{Dry mass of unpretreated solids}}* \% \;{\text{of glucan in unpretreated solids}}} \right)$$

$${\text{Enzyme loading}} = {\text{mg of protein pergram of glucan in enzymatic hydrolysis flask}}/{\text{glucan yield after pretreatment}}$$

The mass of anhydrous sugar in enzymatic hydrolysis substrates was converted to the mass of the corresponding hydrous form by dividing cellobiose values by 0.95, glucan values by 0.90, and xylan values by 0.88 to compensate for the mass of water added during hydrolysis.

$${\text{Enzymatic}}\left( {\text{Stage 2}} \right){\text{glucose yield}},\% = 100*\left( {{\text{Concentration of monomeric sugar measured by HPLC}}*{\text{total reaction volume of enzymatic hydrolysis flask}}} \right)/\left( {{\text{Mass of glucan in enzymatic hydrolysis flask}}/{\text{anhydrous correction factor}}} \right)$$

For enzymatic hydrolysis samples, average sugar yield was calculated using values for triplicates. Standard deviation values were calculated using the following formula:

$${\text{Standard deviation}} = \sqrt {\frac{{\sum {\left( {x - \overline{x} } \right)^{2} } }}{{\left( {n - 1} \right)}}}$$

## Additional file

**Additional file 1: Table S1.** Furfural concentrations in liquid hydrolyzates after DSA and CELF pretreatment of Alamo switchgrass at varying conditions. **Figure S1.** Flow diagram of pretreatment and enzymatic hydrolysis of switchgrass visualizing Stage 1 and Stage 2. **Figure S2.** Alamo switchgrass (i) before knife milling, (ii) after milling to < 2 mm, (iii) and after milling to < 1 mm.

## Data Availability

The datasets supporting the conclusions of this article are included within the article and its additional file.

## Abbreviations

DSA: dilute sulfuric acid
CELF: Co-solvent Enhanced Lignocellulosic Fractionation
THF: tetrahydrofuran
HPLC: high-performance liquid chromatography
