# Supplementary material for: CELF significantly reduces milling requirements and improves soaking effectiveness for maximum sugar recovery of Alamo switchgrass over dilute sulfuric acid pretreatment
Source: Biotechnol Biofuels. 2019 Jul 10;12:177. doi: 10.1186/s13068-019-1515-7 (PMC6617576; doi:10.1186/s13068-019-1515-7)
Supplement: Supplementary file 1 — Additional file 1: Table S1. Furfural concentrations in liquid hydrolyzates after DSA and CELF pretreatment of Alamo switchgrass at varying conditions. Figure S1. Flow diagram of pretreatment and enzymatic hydrolysis of switchgrass visualizing Stage 1 and Stage 2. Figure S2. Alamo switchgrass (i) before knife milling, (ii) after milling to < 2 mm, (iii) and after milling to < 1 mm. [file 13068_2019_1515_MOESM1_ESM.docx]

**Table S1.** Furfural concentrations in liquid hydrolyzates after DSA and CELF pretreatment of Alamo switchgrass at varying conditions.

| Pretreatment | Conditions | Hydrolyzate furfural concentration |
| --- | --- | --- |
| DSA | 150 **°**C, 20 min | < 0.1 g/L |
|  | 150 **°**C, 30 min | < 0.1 g/L |
|  | 150 **°**C, 40 min | < 0.1 g/L |
|  | 150 **°**C, 50 min | < 0.1 g/L |
|  | 150 **°**C, 60 min | < 0.1 g/L |
|  | 160 **°**C, 20 min | < 0.1 g/L |
|  | 160 **°**C, 30 min | 0.22 g/L |
|  | 160 **°**C, 40 min | 0.76 g/L |
|  | 160 **°**C, 50 min | 0.92 g/L |
|  | 160 **°**C, 60 min | 1.62 g/L |
| CELF | 140 **°**C, 20 min | < 0.1 g/L |
|  | 140 **°**C, 30 min | < 0.1 g/L |
|  | 140 **°**C, 40 min | 0.56 g/L |
|  | 140 **°**C, 50 min | 0.89 g/L |
|  | 150 **°**C, 10 min | < 0.1 g/L |
|  | 150 **°**C, 20 min | < 0.1 g/L |
|  | 150 **°**C, 25 min | < 0.1 g/L |
|  | 150 **°**C, 30 min | 0.11 g/L |

**Figure S1.** Flow diagram of pretreatment and enzymatic hydrolysis of switchgrass visualizing Stage 1 and Stage 2.


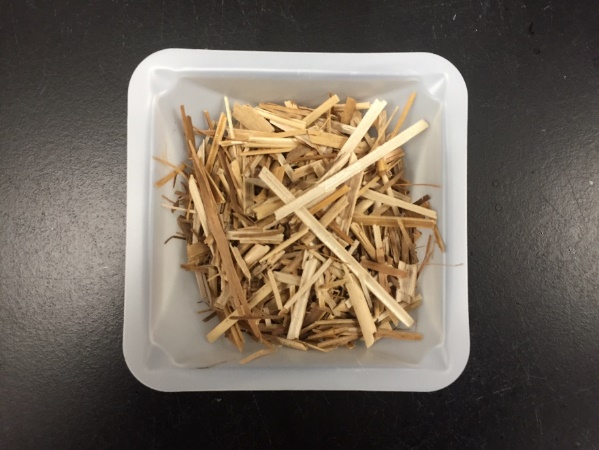

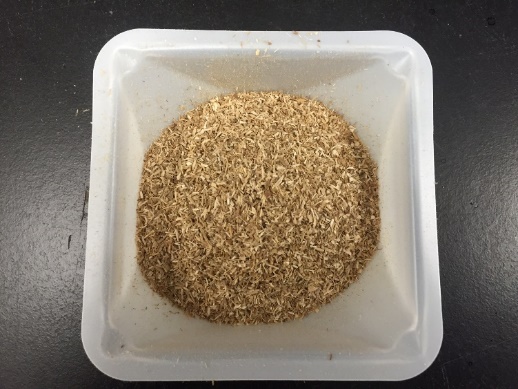

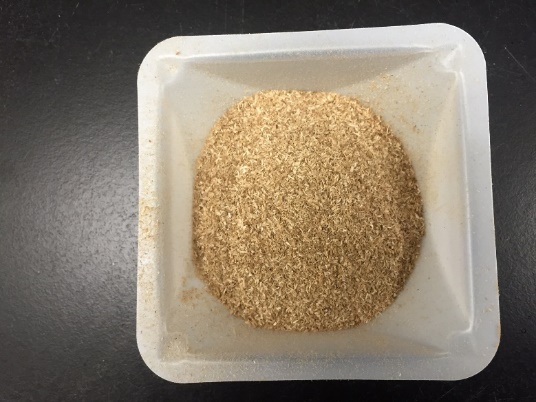


(i)

(ii)

(iii)

**Figure S2.** Alamo switchgrass (i) before knife milling, (ii) after milling to < 2 mm, (iii) and after milling to < 1 mm.
